# Comparative evaluation of the accuracy and reliability of ChatGPT versions in providing information on *Helicobacter pylori* infection

**DOI:** 10.3389/fpubh.2025.1566982

**Published:** 2025-05-15

**Authors:** Yi Ye, En-dian Zheng, Qiao-li Lan, Le-can Wu, Hao-yue Sun, Bei-bei Xu, Ying Wang, Miao-miao Teng

**Affiliations:** ^1^Department of Gastroenterology, Wenzhou People's Hospital, The Wenzhou Third Clinical Institute Affiliated to Wenzhou Medical University, Wenzhou, China; ^2^Postgraduate Training Base Alliance of Wenzhou Medical University, Wenzhou, China

**Keywords:** artificial intelligence, *Helicobacter pylori*, large language model, patient education, ChatGPT

## Abstract

**Objective:**

This study aimed to evaluate the accuracy and reliability of responses provided by three versions of ChatGPT (ChatGPT-3.5, ChatGPT-4, and ChatGPT-4o) to questions related to *Helicobacter pylori* (Hp) infection, as well as to explore their potential applications within the healthcare domain.

**Methods:**

A panel of experts compiled and refined a set of 27 clinical questions related to Hp. These questions were presented to each ChatGPT version, generating three distinct sets of responses. The responses were evaluated and scored by three gastroenterology specialists utilizing a 5-point Likert scale, with an emphasis on accuracy and comprehensiveness. To assess response stability and reliability, each question was submitted three times over three consecutive days.

**Results:**

Statistically significant differences in the Likert scale scores were observed among the three ChatGPT versions (*p* < 0.0001). ChatGPT-4o demonstrated the best performance, achieving an average score of 4.46 (standard deviation 0.82) points. Despite its high accuracy, ChatGPT-4o exhibited relatively low repeatability. In contrast, ChatGPT-3.5 exhibited the highest stability, although it occasionally provided incorrect answers. In terms of readability, ChatGPT-4 achieved the highest Flesch Reading Ease score of 24.88 (standard deviation 0.44), however, no statistically significant differences in readability were observed among the versions.

**Conclusion:**

All three versions of ChatGPT were effective in addressing Hp-related questions, with ChatGPT-4o delivering the most accurate information. These findings suggest that artificial intelligence-driven chat models hold significant potential in healthcare, facilitating improved patient awareness, self-management, and treatment compliance, as well as supporting physicians in making informed medical decisions by providing accurate information and personalized recommendations.

## 1 Introduction

*Helicobacter pylori* (Hp) is one of the most prevalent pathogens worldwide, associated with several gastrointestinal disorders, including peptic ulcers, gastric marginal zone lymphoma, and gastric cancer (1). The emergence of antibiotic resistance in Hp has risen significantly in recent years, attributable to limited therapeutic options, the widespread use of certain antibiotics in the general population, and the pathogen's unique adaptive mechanisms (2). Consequently, the success rate of eradication therapy has declined, placing a significant burden on regional healthcare systems (3, 4).

Previous studies have underscored that the importance of monitoring medication adherence and educating patients about treatment compliance (5, 6). A recent survey conducted in the United States revealed that the general population possesses limited knowledge about Hp, indicating poor adherence to treatment protocols (7). Notably, 81% of respondents indicated that understanding the link between Hp and gastric cancer would encourage greater adherence to treatment regimens. Improving awareness of Hp and fostering compliance with treatment could mitigate antibiotic resistance and enhance eradication success rates. Concurrently, an increasing number of people prefer obtaining health information from the Internet, making it an effective tool for disseminating health knowledge and enhancing treatment compliance (8, 9).

With advancements in big data and artificial intelligence (AI) technologies, the use of natural language dialogue systems, such as ChatGPT has gained popularity for accessing medical knowledge (10). ChatGPT, a Generative Pre-Trained Transformer model developed by OpenAI, is a natural language processing (NLP) model that leverages deep learning techniques to comprehend and generate human-like text. Its multilingual capabilities enable it to perform diverse tasks such as answering questions, providing information, writing code, and translating text. Specifically, in the medical field, ChatGPT holds potential for applications such as supporting clinical diagnoses, generating radiology reports, and drafting medical notes (11, 12). Research has found that ChatGPT-4 surpasses Google's Bard in reliability, accuracy, and stability when responding to patient inquiries (13).

AI-driven question-answering systems including ChatGPT, are specifically designed to provide direct and comprehensible responses, making them effective for providing the public with specific and actionable health advice rather than general professional medical interpretations (14). However, it remains essential for clinicians and healthcare/medical communicators to verify the accuracy of information generated by AI-based models to ensure reliability and safety.

This study aimed to evaluate the accuracy, reproducibility, and potential applications of ChatGPT versions (ChatGPT-3.5, ChatGPT-4, and ChatGPT-4o) in responding to questions related to Hp. Additionally, it sought to verify their role in the dissemination of healthcare information.

## 2 Methods and materials

### 2.1 Methods

Forty questions were initially formulated by an expert panel, guided by the most recent recommendations for managing Hp infection as outlined in the Maastricht VI/Florence consensus report and established practices in Hp diagnosis and treatment (15). These questions were subsequently reviewed and refined by a secondary panel of experts to eliminate redundancies and ambiguities. Efforts were made to phrase the questions in plain, everyday language to better align with how the public seeks medical information. Ultimately, 27 questions were finalized, categorized into five distinct domains (Appendix 1): prevention (*n* = 5), patient education (*n* = 5), diagnosis (*n* = 5), disease management (*n* = 6), and treatment (*n* = 6).

**Figure 1:**
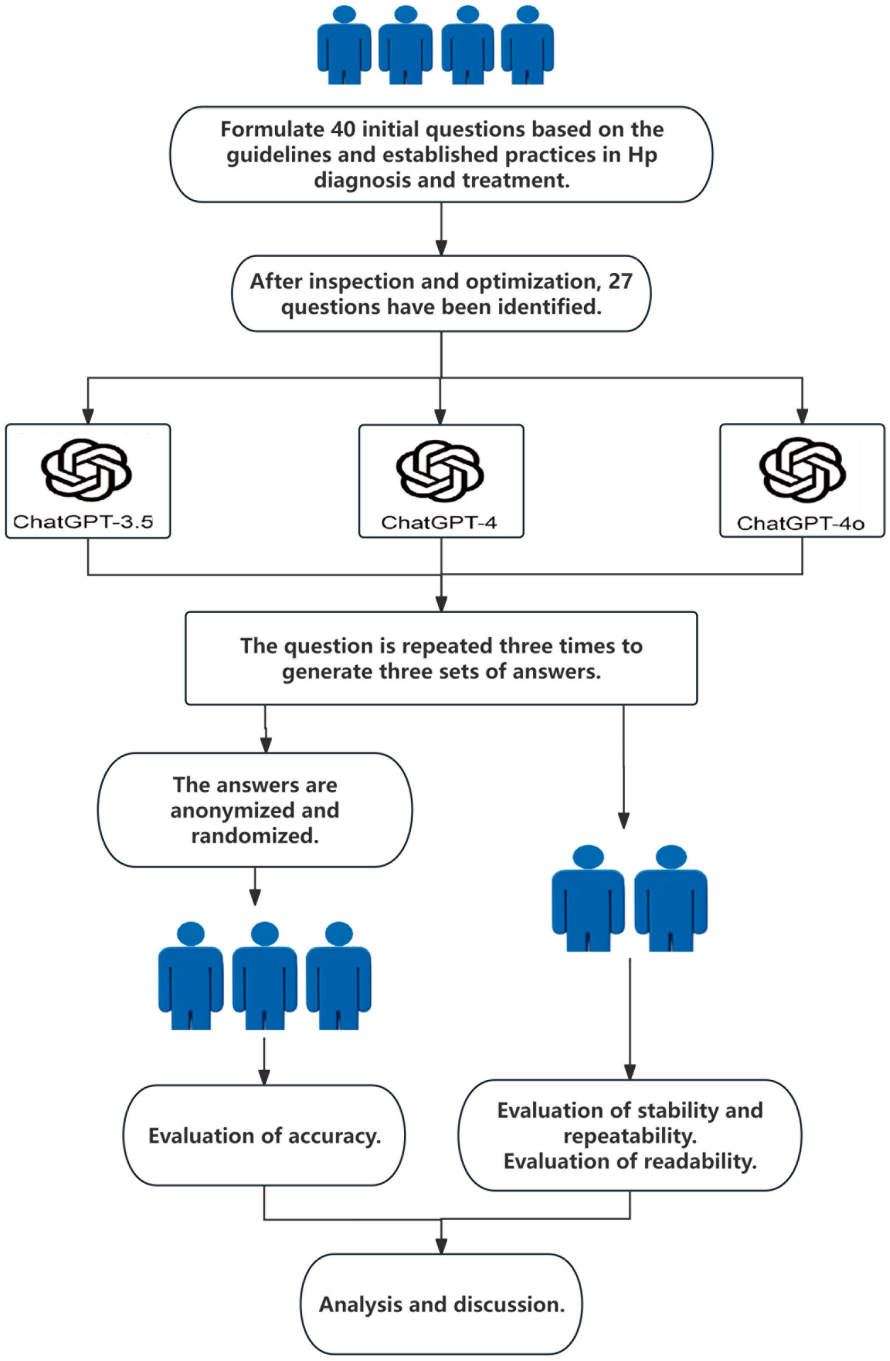
Flowchart of overall study design.

This study was approved by the Ethics Committee of Wenzhou People's Hospital (No.KY-202503-038). The 27 clinical questions related to Hp were entered into ChatGPT-3.5, ChatGPT-4, and ChatGPT-4o using default parameter settings, resulting in three sets of documented responses (Figure 1). To evaluate reproducibility, each question was submitted to each version of ChatGPT three times between July 12 and July 14, 2024, while maintaining the same parameter settings. Each set of responses was independently evaluated by three experienced gastroenterologists. The three gastroenterologists evaluated and scored the responses using a 5-point Likert scale, with scores of 4 or 5 indicating accurate and comprehensive responses, and scores of 1–3 indicating incorrect or incomplete responses (16).

Additionally, readability was assessed using the Flesch Reading Ease (FRE) (17)and Flesch-Kincaid Grade Level (FKGL) (18) metrics. The FRE score, ranging from 0 to 100, measures text readability, with higher scores indicating easier comprehension. For example, a score of 90–100 is easily understandable by an average 11-year-old, while a score of 0–30 is considered difficult and best understood by university graduates. The FKGL score, on the other hand, corresponds to the U.S. grade level required to understand the text, with lower scores indicating simpler language. For instance, a score of 8.0 means the text is understandable by an eighth grader.

Stability and reproducibility were assessed independently by two experts. All experts, including the three gastroenterologists mentioned earlier, were from our center and all had more than 10 years of experience in diagnosing and treating Hp. Responses were categorized as “basically the same” if three responses to each question had 75% or more content repetition, “not exactly the same” if responses had <75% content repetition, and “incorrect” if any response contained misinformation or lacked comprehensiveness.

### 2.2 Statistical analysis

Continuous variables were presented as mean and standard deviation (SD). Comparisons across multiple groups were performed using one-way analysis of variance (ANOVA), with *a p*-value of <0.05 considered statistically significant. Kendall's coefficient was calculated to assess the level of agreement among the three experts regarding the Likert scale ratings. All statistical analyses were conducted using SPSS, Version 27.0 (IBM) and GraphPad Prism, Version 10.1.2 (GraphPad Software, Inc.).

## 3 Results

### 3.1 Evaluation of accuracy

**Figure 2:**
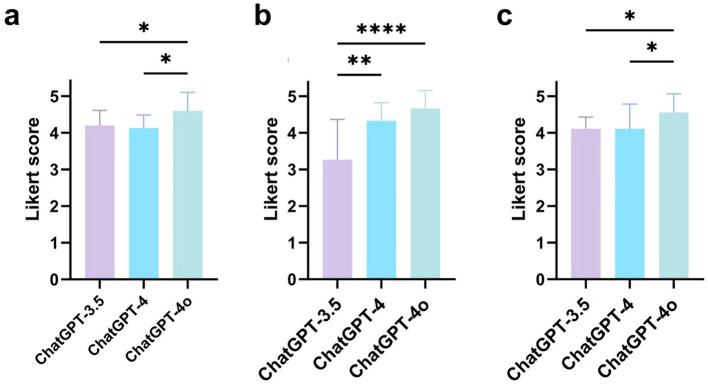
Comparison of average Likert scores between different versions of ChatGPT across three clinical domains: **(a)** Prevention; **(b)** Diagnosis; **(c)** Disease management (*p < 0.05, **p < 0.01, ****p < 0.0001).

The Likert scale scores for each AI model are presented in Table 1. A statistically significant difference (*p* < 0.0001) was observed among the three versions of ChatGPT. ChatGPT-4o demonstrated the best performance, achieving an average score of 4.46 (SD 0.82) points. Detailed Likert scale results for each response are provided in Appendix 2. Subgroup analysis across the five domains demonstrated that ChatGPT-4o achieved the highest average Likert scores in the categories of prevention, diagnosis, and disease management (Figure 2). Kendall's coefficient was calculated as 0.845 (0.790–0.898), indicating a high degree of consistency among the evaluators.

**Table 1:** Readability evaluation results.

**Readability**	**ChatGPT-3.5, mean (SD)**	**ChatGPT-4.0, mean (SD)**	**ChatGPT-4o, mean (SD)**	***P*-value**
Word count	155.5(67.09)	199.2(75.51)	230.9(104.5)	0.006
Flesch Reading Ease^a^	20.24(9.44)	24.88(8.04)	21.64(10.54)	0.184
Flesch-Kincaid Grade Level^b^	15.19(1.60)	14.82(1.48)	15.01(2.09)	0.736

^*a*^Flesch Reading Ease is based on average sentence length and word complexity. It gives a text a score between 0 and 100, with higher scores indicating easier to read.

^*b*^Flesch-Kincaid Grade Level gives a text a score according to the grade level of the United States, with higher scores indicating higher requirement for reading.

### 3.2 Evaluation of readability

The average word count, FRE, and FKGL scores for responses are summarized Table 2. ChatGPT-4o generated the longest responses, with an average word count of 230.9 (SD 104.5), followed by ChatGPT-4. Although ChatGPT-4 demonstrated superior FRE and FKGL scores compared to the other two models, no statistical difference in readability metrics was observed across the three versions.

**Table 2:** Comparisons of average Likert scores among 3 versions of ChatGPT.

**Question categories**	**ChatGPT-3.5, mean (SD)**	**ChatGPT-4.0, mean (SD)**	**ChatGPT-4o, mean (SD)**	***P*-value**
All questions	3.94(0.75)	4.14(0.75)	4.494(0.74)	<0.001
Prevention	4.20(0.41)	4.13(0.35)	4.600(0.51)	<0.001
Patient education	4.13(0.35)	4.33(0.82)	4.400(0.51)	0.444
Diagnosis	3.267(1.10)	4.33(0.49)	4.667(0.49)	<0.001
Disease management	4.11(0.32)	4.11(0.68)	4.556(0.51)	0.019
Treatment	3.94(0.87)	3.83(1.10)	4.278(1.28)	0.453

### 3.3 Evaluation of stability and repeatability

The stability analysis, presented in Table 3, indicates that ChatGPT-3.5 demonstrated the highest stability, with 81.5% of its responses remaining “basically the same.” However, it also produced the highest proportion of incorrect responses (11.1%). In contrast, ChatGPT-4o exhibited the lowest repeatability, with only 48.1% of its responses categorized as “basically the same” and another 48.1% as “not exactly the same.” Despite this lower repeatability, ChatGPT-4o achieved the highest accuracy, with only one set of answers deemed “incorrect.”

**Table 3:** Stability evaluation results.

**Stability**	**ChatGPT-3.5, *n* (%)**	**ChatGPT-4.0, *n* (%)**	**ChatGPT-4o, *n* (%)**
Basically the same	22(81.5)	20(74.1)	13(48.1)
Not exactly the same	2(7.4)	4(14.8)	13(48.1)
Incorrect	3(11.1)	3(11.1)	1(3.7)

## 4 Discussion

This study represents the first to evaluate the accuracy and readability of different versions of ChatGPT in answering Hp-related questions. The findings highlight the significant potential of ChatGPT as an AI-driven question-answering tool in the healthcare sector. The three AI models displayed notable variations in their ability to accurately respond to Hp-related clinical questions, with ChatGPT-4o demonstrating the best performance. Subgroup analysis in the areas of disease prevention, diagnosis, and management indicated that ChatGPT-4o consistently delivered the most accurate information compared to the other two models. Although no significant differences in readability were observed among the three versions of ChatGPT, ChatGPT-3.5 produced the most succinct answers, while ChatGPT-4 and ChatGPT-4o provided longer, more comprehensive responses.

The ChatGPT-4o model exhibited greater complexity and advanced features compared to earlier versions; however, its responses demonstrated lower reproducibility, as responses to the same question varied across different instances. Conversely, ChatGPT-3.5, while simpler in design, demonstrated the highest reproducibility. The variability in ChatGPT-3.5′s scores in the diagnosis domain may stem from the technical nature of diagnostic information. For example, in response to the question, “How can I be sure that Hp has been completely eradicated after treatment?”, ChatGPT incorrectly suggested that symptom relief could indicate successful treatment, which led to lower scores.

The declining success rates of eradication therapies and the growing issue of antibiotic resistance in Hp necessitate the implementation of effective measures (19). Hp infection is primarily acquired in childhood (20), with a disproportionate disease burden observed in populations with limited resources. Infection rates are notably higher in developing countries, where socioeconomic challenges persist (21).Socioeconomic improvements have been shown to play a significant role in reducing infection rates (22).

ChatGPT demonstrates significant potential as a platform for disseminating Hp-related information to the public, covering critical topics such as diagnosis, treatment, and follow-up care. Its ability to serve as a repository for clinical recommendations and guidelines enables it to deliver personalized health advice and reminders, which could improve patient adherence to Hp treatment regimens. Specifically, ChatGPT can assist in standardizing patient education by providing consistent and evidence-based information on Hp prevention, diagnostic methods, and therapeutic options. This is particularly valuable in regions with limited access to specialized healthcare providers. Additionally, ChatGPT's multilingual capabilities and accessibility make it a powerful tool for bridging language barriers and expanding the reach of Hp-related research and education globally.

In broader terms, ChatGPT holds promise for enhancing patient access to medical knowledge, reducing healthcare costs, and contributing to the equitable distribution of medical resources. By facilitating real-time, on-demand access to accurate medical information, ChatGPT can support clinicians in staying updated with the latest Hp research and guidelines, thereby improving diagnostic accuracy and treatment outcomes. Furthermore, its potential to analyze and summarize large volumes of medical literature could accelerate Hp research by identifying trends, gaps, and emerging therapies. The widespread use of ChatGPT could also alleviate the economic burden on national healthcare systems by reducing unnecessary consultations and optimizing resource allocation.

A growing body of evidence supports the utility of ChatGPT as a medical question-answering systems, including its application in Hp-related contexts (13, 23, 24). Consistent with the findings of this study, Lai et al. examined the accuracy and reproducibility of ChatGPT-3.5 in answering Hp-related questions and determined that it could provide correct answers to most Hp-related queries (25). Compared to previous studies (24, 25), we evaluated the capability of the latest state-of-the-art ChatGPT model (ChatGPT-4o) in providing information related to Hp. The GPT-4o demonstrates significant improvements in real-time processing and reasoning capabilities, enabling faster and more accurate responses to users' complex queries and needs. Additionally, we compared the answer length and readability across three ChatGPT versions, as these factors directly impact the efficiency of medical information access and comprehension for non-expert users—an aspect that has not been thoroughly explored in prior research.

However, limitations remain, even in advanced models. For instance, when prompted about treatment options for patients with penicillin allergies, both ChatGPT-4 and ChatGPT-4o recommended regimens containing amoxicillin. Although some responses did mention that metronidazole could be an alternative for patients allergic to amoxicillin, these inconsistencies highlights the need for careful evaluation of AI-generated medical information. Given the potential for ChatGPT-generated content to directly impact the health and wellbeing of patients, it is crucial to ensure the absolute accuracy of the information provided. This accuracy is essential for assisting users in obtaining reliable disease-related knowledge and information, thereby preventing any potential harm. However, ChatGPT's tendency to present responses with a high degree of confidence, even when the information provided is inaccurate (26), raises significant legal concerns, particularly when inappropriate medical advice is provided in real-life situations (27). Therefore, it is imperative to approach the use of AI systems with caution.

Additionally, the use of ChatGPT in healthcare poses specific risks and limitations, particularly concerning patient privacy (28). When individuals seek health-related information through ChatGPT, the system may collect and store sensitive personal data, such as medical history, examination results, diagnostic results, and other private health information. These practices raise significant concerns regarding information security, making it crucial for regulatory authorities to supervise, evaluate, and approve the use of ChatGPT in specific healthcare applications or situations (29).

Second, interpretability is crucial for building trust between users and platforms. However, the limited interpretability of ChatGPT, due to its black-box nature, may raise concerns among users regarding the validity and reliability of solutions it provides (29). Third, the reproducibility of ChatGPT's responses is another critical issue, as its outputs can vary across different times or conditions, resulting in inconsistent and unreliable outcomes in healthcare practice (30). Moreover, ChatGPT's performance is highly influenced by the phrasing and structure of the questions posed. Non-medical professionals, who may lack the expertise to formulate standardized and precise queries, could face challenges in optimizing the model's effectiveness. To address this limitation, the questions in this study were intentionally framed using everyday language and expressions rather than medical terminology. This approach aimed to create a more relatable application scenario, enhancing the accessibility and relevance of ChatGPT for the general public.

However, this study has some limitations. First, while efforts were made to simulate practical application scenarios by framing questions in everyday language, these interactions did not fully replicate real-world exchanges between patients and ChatGPT. Future research should incorporate real-world patient interactions to better evaluate the model's performance. Second, the absence of standardized guidelines for evaluating ChatGPT's performance may contribute to variability in evaluation outcomes, particularly for open-ended questions, as subjective interpretations among physicians could differ.

## 5 Conclusion

This study underscores the immense potential of ChatGPT in addressing Hp-related clinical and public health concerns, with ChatGPT-4o demonstrating superior accuracy and comprehensiveness among the evaluated models. Looking forward, AI-powered conversation models specifically designed for healthcare applications are expected to drive significant advancements. These models hold the potential to improve patient awareness of self-management and treatment compliance, enabling them to better engage and cooperate in their treatment regimen. Additionally, they can serve as valuable tools for physicians, providing accurate and personalized information to facilitate the most favorable clinical decision-making.

## Data Availability

The original contributions presented in the study are included in the article/Supplementary material, further inquiries can be directed to the corresponding author.
