# Supplementary material for: Comparative evaluation of the accuracy and reliability of ChatGPT versions in providing information on Helicobacter pylori infection
Source: Front Public Health. 2025 May 15;13:1566982. doi: 10.3389/fpubh.2025.1566982 (PMC12119545; doi:10.3389/fpubh.2025.1566982)
Supplement: Supplementary file 1 [file Data_Sheet_1.docx]

Appendix 1. Complete list of questions and their Likert scores

| **Clinical Question** | **Likert Score for ChatGPT-3.5** | | | | **Likert Score for ChatGPT-4** | | | **Likert Score for ChatGPT-4o** | | |
| --- | --- | --- | --- | --- | --- | --- | --- | --- | --- | --- |
| **Prevention** | **R 1** | **R 2** | **R 3** | **R 1** | | **R 2** | **R 3** | **R 1** | **R 2** | **R 3** |
| What is Helicobacter pylori? | 4 | 4 | 4 | 4 | | 4 | 4 | 4 | 4 | 4 |
| How can I prevent H. pylori infection? | 5 | 5 | 5 | 4 | | 4 | 4 | 4 | 4 | 4 |
| Is it possible for family members to become infected if someone in the family has H. pylori? | 4 | 4 | 4 | 4 | | 4 | 4 | 5 | 5 | 5 |
| I was wondering if there is currently a vaccine for H. pylori? | 4 | 4 | 4 | 5 | | 4 | 4 | 5 | 5 | 5 |
| What conditions facilitate the infection of Helicobacter pylori? | 4 | 4 | 4 | 5 | | 4 | 4 | 5 | 5 | 5 |
| **Patient education** | | | | | | | | | | |
| What are the risks associated with non-steroidal inflammatory drug use after bariatric surgery? | 4 | 4 | 4 | 5 | | 5 | 5 | 4 | 4 | 4 |
| Can Helicobacter pylori infection be self-healed? | 5 | 4 | 5 | 5 | | 4 | 5 | 5 | 4 | 4 |
| What are the symptoms associated with Helicobacter pylori infection? | 4 | 4 | 4 | 3 | | 3 | 3 | 5 | 5 | 5 |
| What are the risks of getting infected with Helicobacter pylori? | 4 | 4 | 4 | 4 | | 4 | 4 | 5 | 5 | 4 |
| Is there a link between getting stomach cancer and being infected with Helicobacter pylori? | 4 | 4 | 4 | 5 | | 5 | 5 | 4 | 4 | 4 |
| **Diagnosis** | | | | | | | | | | |
| What are the methods for diagnosis of Helicobacter pylori infection? | 4 | 4 | 4 | 4 | | 4 | 4 | 5 | 5 | 5 |
| How can I be sure that HP has been completely eliminated after the treatment? | 1 | 1 | 2 | 4 | | 4 | 4 | 4 | 4 | 4 |
| How long should we allow for review after Helicobacter pylori eradication treatment? | 4 | 4 | 4 | 5 | | 5 | 5 | 4 | 5 | 4 |
| Who should be tested for H. pylori? | 3 | 3 | 3 | 4 | | 4 | 4 | 5 | 5 | 5 |
| What's the best way to screen for a Helicobacter pylori infection? | 4 | 4 | 4 | 5 | | 5 | 4 | 5 | 5 | 5 |
| **Disease management** | | | | | | | | | | |
| Can I get reinfected with Helicobacter pylori after being cured? | 4 | 4 | 4 | 4 | | 4 | 4 | 5 | 4 | 4 |
| What should I do if my child is infected with Helicobacter pylori? | 4 | 5 | 5 | 4 | | 4 | 4 | 5 | 5 | 5 |
| I'm wondering if eradicating Helicobacter pylori can reverse gastric atrophy？ | 4 | 4 | 4 | 5 | | 5 | 4 | 5 | 4 | 5 |
| Should patients on long-term aspirin use get tested for and treated for H. pylori? | 4 | 4 | 4 | 3 | | 3 | 3 | 5 | 4 | 5 |
| Is treatment necessary for all patients with a Helicobacter pylori infection? | 4 | 4 | 4 | 5 | | 5 | 5 | 4 | 5 | 5 |
| I'm wondering if there's a link between Helicobacter pylori infection and an increased risk of gastrointestinal bleeding in patients taking anticoagulants. | 4 | 4 | 4 | 4 | | 4 | 4 | 4 | 4 | 4 |
| **Treatment** | | | | | | | | | | |
| Do probiotics work for the treatment of H. pylori eradication? | 5 | 5 | 5 | 4 | | 4 | 4 | 5 | 5 | 5 |
| I'm currently being treated for H. pylori. Is it okay for me to drink alcohol while I'm on this course of treatment? | 4 | 4 | 4 | 5 | | 5 | 4 | 5 | 5 | 5 |
| How to treat Helicobacter pylori in patients with penicillin allergy? | 4 | 5 | 4 | 1 | | 2 | 2 | 1 | 2 | 2 |
| How long do I need to take the medication for H. pylori? | 4 | 4 | 4 | 4 | | 4 | 4 | 4 | 4 | 4 |
| I'm looking for advice on how to treat a Helicobacter pylori infection. | 4 | 4 | 4 | 4 | | 4 | 4 | 5 | 5 | 5 |
| What are the side effects of drugs used to treat Helicobacter pylori? | 3 | 2 | 2 | 5 | | 5 | 4 | 5 | 5 | 5 |

R :Reviewer

**Appendix 2. Three ChatGPT Versions of Answers to Individual Questions**

| **Clinical Question** | **ChatGPT-3.5** | **ChatGPT-4** | **ChatGPT-4o** |
| --- | --- | --- | --- |
| **Prevention** | | | |
| How Helicobacter pylori is transmitted？ | Helicobacter pylori is primarily transmitted through person-to-person contact. The exact route of transmission is not fully understood, but it is believed to occur through:  1.Oral-Oral Transmission: This is thought to be the most common route. H. pylori can be passed from person to person through direct contact with saliva, such as kissing or sharing utensils.  2.Fecal-Oral Transmission:H. pylori can also spread through fecal contamination of food, water, or objects. This can happen in settings where hygiene is poor or where there is inadequate sanitation.  3.Gastro-Oral Transmission:Some research suggests that the bacterium can be transmitted from the stomach to the mouth, possibly through regurgitation or vomiting, and then to another person.  It's important to note that while these are the suspected routes of transmission, the exact mechanisms can vary, and not everyone exposed to H. pylori will necessarily become infected. | Helicobacter pylori, commonly known as H. pylori, is a bacterium that can live in the digestive tract and is responsible for many cases of stomach ulcers and some types of gastritis. It's primarily transmitted through direct contact with saliva, vomit, or fecal matter from an infected person. This can happen in several ways:  1.Oral-oral transmission: This mode of transmission can occur when there is an exchange of oral fluids, such as through kissing or sharing eating utensils, drinking glasses, or toothbrushes.  2.Fecal-oral transmission: This can happen through consuming food or water that's contaminated with fecal matter containing the bacteria. This type of transmission is more common in areas with poor sanitation.  3. Gastro-oral transmission: This involves the transfer of stomach contents to the mouth, potentially spreading to others through direct or indirect contact.  Good hygiene practices, such as regular handwashing and proper food preparation, are key to preventing the spread of H. pylori. | Helicobacter pylori (H. pylori) is a type of bacteria that can infect the stomach and is known to be a significant cause of peptic ulcers and chronic gastritis. The transmission of H. pylori primarily occurs through the following routes:  1. Person-to-Person Contact: This is the most common mode of transmission. H. pylori can spread through direct contact with saliva, vomit, or fecal matter of an infected person. Close personal contact, such as kissing or sharing eating utensils, can facilitate the transfer of the bacteria.  2. Fecal-Oral Route: Poor hygiene practices, particularly in areas with inadequate sanitation, can lead to the spread of H. pylori. Contaminated food or water can carry the bacteria, and if ingested, it can lead to infection.  3.Oral-Oral Route: There is evidence to suggest that the bacteria can be transmitted from mouth to mouth through activities such as sharing drinks, eating utensils, or through oral contact.  4.Environmental Factors: H. pylori can survive for short periods outside the human body in certain environments. Contaminated surfaces, especially in communal settings like schools or daycare centers, can be a source of infection if proper hygiene is not maintained.  Preventive measures to reduce the risk of H. pylori infection include practicing good hygiene, such as thorough handwashing with soap and water, drinking clean and safe water, and ensuring food is properly prepared and cooked. |
| **Patient education** | | | |
| What are the symptoms associated with Helicobacter pylori infection? | Helicobacter pylori infection can manifest with various symptoms, although many infected individuals may not experience any symptoms at all. When symptoms do occur, they can include:  1. Abdominal pain: This pain is often described as a dull or burning sensation in the upper abdomen, which may occur between meals or during the night.  2. Nausea and vomiting: Some people with H. pylori infection may experience nausea and occasional vomiting.  3. Bloating: Abdominal bloating or a feeling of fullness may occur, especially after meals.  4. Loss of appetite: A reduced desire to eat can accompany H. pylori infection, possibly due to discomfort after eating.  5. Frequent burping: Excessive belching or burping may occur, often due to increased gas production in the stomach.  6. Unintentional weight loss: Some individuals may lose weight without trying, which can be a result of decreased appetite or other digestive issues.  7. Fatigue: Generalized weakness or fatigue may be present, although this is less commonly associated with H. pylori infection compared to other symptoms.  It's important to note that these symptoms can overlap with other gastrointestinal conditions, so a proper medical diagnosis is essential for accurate treatment. | Helicobacter pylori (H. pylori) infection can lead to various symptoms, primarily affecting the stomach and gastrointestinal tract. Here are some common symptoms associated with this infection:  1. Abdominal Pain or Discomfort: Often described as a burning or gnawing feeling in the stomach, typically when the stomach is empty.  2. Bloating and Fullness: Feeling full after eating only a small amount of food and experiencing uncomfortable bloating.  3. Nausea: Some people may experience nausea, although it might not always lead to vomiting.  4. Frequent Burping: Increased burping can be another symptom of the infection.  5. Loss of Appetite: There may be a decrease in appetite, which can sometimes be accompanied by weight loss.  6. Heartburn: A burning sensation in the chest, often after eating or at night.  It's important to note that many people infected with H. pylori do not experience any symptoms. However, this bacterium can cause conditions such as gastritis (inflammation of the stomach lining) and peptic ulcers, which can lead to more severe symptoms like bleeding ulcers, leading to black or tarry stools, or severe, persistent abdominal pain. If you or someone you know is experiencing these symptoms, it's advisable to consult a healthcare provider for proper diagnosis and treatment. | Helicobacter pylori (H. pylori) infection can cause a range of symptoms, although some individuals may be asymptomatic. Common symptoms associated with H. pylori infection include:  1. Abdominal Pain: A burning or gnawing pain in the stomach, often located in the upper abdomen. This pain may be more noticeable when the stomach is empty.  2. Bloating: A feeling of fullness or swelling in the abdomen.  3. Nausea and Vomiting: These symptoms can occur particularly after eating.  4. Loss of Appetite: A reduced desire to eat, which can sometimes lead to weight loss.  5. Frequent Burping: An increase in belching or burping can occur.  6. Indigestion (Dyspepsia): General discomfort in the upper abdomen, including a sense of fullness after eating small meals.  7. Gastrointestinal Bleeding: In severe cases, H. pylori infection can cause ulcers that may lead to bleeding, resulting in symptoms like black, tarry stools or vomiting blood.  8. Fatigue: Chronic infection and associated gastrointestinal symptoms can lead to general fatigue and weakness.  9. Anemia: Blood loss from gastrointestinal bleeding can result in anemia, characterized by fatigue, weakness, and pale skin.  If you suspect an H. pylori infection, it is essential to consult a healthcare provider for diagnosis and appropriate treatment. |
| **Diagnosis** | | | |
| Who should be tested for H. pylori? | Testing for H. pylori, a bacterium linked to stomach ulcers and some types of stomach cancer, is typically recommended for individuals who present with symptoms such as persistent abdominal pain, nausea, bloating, or unexplained weight loss. Additionally, testing is considered for those who have a history of peptic ulcers or stomach cancer, or who require long-term use of nonsteroidal anti-inflammatory drugs (NSAIDs) or aspirin.  Furthermore, individuals may also be tested if they have a family history of stomach cancer or if they are undergoing endoscopy for other reasons. Testing for H. pylori can involve several methods, including blood tests, breath tests, stool tests, or tissue biopsy during endoscopy.  If you have specific concerns or symptoms related to H. pylori infection, consulting with a healthcare provider is advisable to determine whether testing is necessary and which method is most appropriate for your situation. | Testing for Helicobacter pylori (H. pylori) is recommended for individuals who have conditions or symptoms that may be linked to infection with the bacteria. Here are some of the common scenarios where testing is advised:  1. Symptoms of Peptic Ulcer Disease: Individuals experiencing symptoms such as stomach pain, nausea, bloating, or other signs of ulcers may be tested for H. pylori, as it is a major cause of ulcers.  2. History of Peptic Ulcer: Anyone who has a history of peptic ulcers should be tested for H. pylori because it can increase the risk of recurrence if not treated.  3. Active Gastric or Duodenal Ulcers: If someone is diagnosed with an active ulcer, testing for H. pylori can determine if it is the underlying cause, which would influence the treatment approach.  4. MALT Lymphoma: This is a type of stomach cancer associated with H. pylori infection. Testing is part of the management and treatment of this condition.  5. After Gastric Cancer Surgery: Testing might be recommended for monitoring purposes after treatment for early gastric cancer.  6. Unexplained Iron Deficiency Anemia: When anemia cannot be explained by other causes, testing for H. pylori might be suggested, as it can lead to chronic stomach inflammation that affects iron absorption.  7. Adults with Chronic Dyspepsia: For persistent unexplained dyspepsia (indigestion), testing for H. pylori can be useful to determine if it's the cause of the symptoms.  8. Family History of Gastric Cancer: Individuals with a family history of gastric cancer may be advised to undergo testing for H. pylori as part of preventive strategies.  The decision to test for H. pylori should be made in consultation with a healthcare provider, who can recommend the most appropriate testing method based on the individual’s symptoms and medical history. | Testing for Helicobacter pylori (H. pylori) is recommended for individuals in several specific situations:  1. Active Peptic Ulcer Disease: Individuals with active peptic ulcer disease (gastric or duodenal ulcers) should be tested for H. pylori, as it is a common cause of these conditions.  2. History of Peptic Ulcer Disease: Those with a documented history of peptic ulcer disease who have not been previously tested or treated for H. pylori.  3. Gastric Mucosa-Associated Lymphoid Tissue (MALT) Lymphoma: Patients diagnosed with this type of lymphoma should be tested, as H. pylori eradication can sometimes lead to remission.  4. Dyspepsia: Individuals under 60 years old with dyspeptic symptoms without alarm features (such as unintended weight loss, anemia, gastrointestinal bleeding, or difficulty swallowing) may be tested for H. pylori.  5. Unexplained Iron Deficiency Anemia: If no other causes for iron deficiency anemia are found, testing for H. pylori may be considered.  6. Idiopathic Thrombocytopenic Purpura (ITP): Patients with this condition should be tested for H. pylori, as eradication of the bacteria can improve platelet counts in some individuals.  7. Family History of Gastric Cancer: Individuals with a first-degree relative who has been diagnosed with gastric cancer may benefit from H. pylori testing.  8. Patients Who Will Be Starting Long-Term NSAID Therapy: Testing for and eradicating H. pylori may be considered before starting long-term nonsteroidal anti-inflammatory drug (NSAID) therapy, as the combination of NSAIDs and H. pylori increases the risk of peptic ulcer disease.  9. Consideration of Gastric Cancer Prevention: In populations at high risk for gastric cancer, such as certain Asian or South American countries, testing and treating H. pylori can be part of a strategy to reduce the incidence of gastric cancer.  These recommendations ensure that testing for H. pylori is targeted towards individuals who are most likely to benefit from diagnosis and treatment, either by resolving symptoms, preventing complications, or addressing underlying conditions associated with H. pylori infection. |
| **Disease management** | | | |
| I'm wondering if eradicating Helicobacter pylori can reverse gastric atrophy？ | Eradicating Helicobacter pylori can potentially reverse gastric atrophy in some cases, especially if it's caught early. Gastric atrophy is often associated with chronic H. pylori infection, which can lead to inflammation and damage to the stomach lining over time. Successful eradication of H. pylori can halt this progression and allow the stomach lining to heal to some extent. However, the extent of reversal depends on factors such as the duration and severity of the atrophy before treatment. It's important to consult with a healthcare professional for personalized advice and management if you suspect gastric atrophy or H. pylori infection. | Eradicating Helicobacter pylori (H. pylori) can potentially have a beneficial impact on gastric atrophy, depending on the stage of the condition at the time of treatment. Gastric atrophy involves the thinning of the stomach lining and a reduction in the number of acid-producing cells, often due to chronic inflammation induced by H. pylori infection.  Here’s how H. pylori eradication might influence gastric atrophy:  1. Early Intervention: If H. pylori is eradicated in the early stages of gastric atrophy, there is a possibility for partial or complete reversal of the condition. Early eradication can reduce inflammation and allow the gastric mucosa a chance to regenerate.  2. Prevention of Progression: In more advanced cases, while the existing damage might not be fully reversible, eradicating H. pylori can help prevent further progression of atrophy and reduce the risk of complications like gastric cancer.  3. Clinical Outcomes: Several studies suggest that the degree of recovery from gastric atrophy post-H. pylori eradication can vary. Some patients may experience significant improvement in their gastric mucosa, while others might see minimal changes if the damage is already severe.  4. Surveillance: After eradication therapy, regular monitoring is recommended, especially for individuals with severe atrophy or other precancerous conditions, to assess the status of the gastric mucosa and manage any ongoing risks.  It's important to consult with a healthcare provider for a tailored treatment approach and follow-up, considering the specifics of each case. | The eradication of Helicobacter pylori (H. pylori) can have significant effects on the stomach's health, but its impact on reversing gastric atrophy varies depending on several factors, including the stage and severity of the atrophy.  1. Early Stages: In the early stages of gastric atrophy, eradicating H. pylori can often lead to partial or complete reversal of the atrophic changes. The removal of the bacteria reduces inflammation and allows the gastric mucosa to heal and regenerate.  2. Advanced Stages: In more advanced stages, where there is significant loss of gastric glands and structural changes, the reversal of gastric atrophy is less likely. Although H. pylori eradication can still reduce inflammation and prevent further progression, the existing damage may not be fully reversible.  3. Prevention of Further Damage: Eradication is crucial in preventing the progression to more severe conditions, such as intestinal metaplasia or gastric cancer. By eliminating the bacteria, you can halt the ongoing damage and reduce the risk of future complications.  4. Overall Health Improvement: Even if complete reversal is not possible, eradicating H. pylori can significantly improve symptoms, reduce the risk of ulcers, and improve the overall health of the stomach lining.  It is essential to consult with a healthcare professional for personalized advice and treatment options. They can provide guidance based on individual health status, the extent of gastric atrophy, and other medical conditions. |
| **Treatment** | | | |
| How to treat Helicobacter pylori in patients with penicillin allergy? | Treating Helicobacter pylori in patients with penicillin allergy typically involves using a combination of antibiotics that do not contain penicillin derivatives. Here are some commonly used treatment regimens:  1. Clarithromycin-based regimen:  - Clarithromycin (500 mg twice daily)  - Metronidazole (500 mg twice daily)  - Proton pump inhibitor (PPI) (e.g., omeprazole, lansoprazole) twice daily  This regimen is taken for 10 to 14 days.  2. Metronidazole-based regimen:  - Metronidazole (500 mg three times daily)  - Tetracycline (500 mg four times daily)  - PPI (twice daily)  This regimen is taken for 10 to 14 days.  3. Bismuth-based quadruple therapy:  - Bismuth subsalicylate (Pepto-Bismol) (525 mg four times daily)  - Metronidazole (500 mg three times daily)  - Tetracycline (500 mg four times daily)  - PPI (twice daily)  This regimen is taken for 10 to 14 days.  It's essential to choose antibiotics based on the patient's specific allergies and medical history. Consultation with a healthcare provider is crucial to tailor the treatment regimen and ensure effective eradication of Helicobacter pylori while managing the penicillin allergy appropriately. | Treating Helicobacter pylori (H. pylori) infection in patients with a penicillin allergy involves using alternative medications that do not include amoxicillin, which is commonly used in standard treatment regimens. Here are some alternative treatment options:  1. Bismuth Quadruple Therapy: This is a recommended first-line treatment for H. pylori in patients allergic to penicillin. It typically includes:  - Bismuth subsalicylate  - Metronidazole  - Tetracycline  - A proton pump inhibitor (PPI) such as omeprazole, esomeprazole, or lansoprazole  This regimen is usually taken for 10-14 days.  2. Concomitant Therapy: This involves the use of four drugs where amoxicillin is replaced by another antibiotic. The regimen includes:  - A PPI  - Clarithromycin  - Metronidazole or tinidazole  - A substitute for amoxicillin such as metronidazole (if not already included)  This treatment also lasts for 10-14 days.  3. Levofloxacin-Based Therapy: This can be used as a second-line treatment and includes:  - A PPI  - Levofloxacin (a fluoroquinolone antibiotic)  - Amoxicillin or, if allergic, metronidazole  Typically, this treatment is given for 10-14 days.  4. Sequential Therapy: This involves two phases of treatment:  - First 5 days: A PPI and metronidazole (instead of amoxicillin)  - Next 5 days: A PPI, clarithromycin, and metronidazole  The total duration of therapy is usually 10 days.  Choosing the best alternative largely depends on the patient’s specific medical history, local antibiotic resistance patterns, and previous exposure to antibiotics. It is crucial to consult with a healthcare provider to tailor the treatment plan according to individual needs and local guidelines. | Treating Helicobacter pylori (H. pylori) infection in patients with a penicillin allergy requires selecting alternative antibiotic regimens that avoid the use of penicillin derivatives. Here are some commonly recommended approaches:  Treatment Regimens  1. Bismuth Quadruple Therapy:  - Bismuth subsalicylate: 300 mg four times daily  - Metronidazole: 500 mg three times daily  - Tetracycline: 500 mg four times daily  - Proton pump inhibitor (PPI): such as omeprazole, 20 mg twice daily  This regimen is often preferred due to its high efficacy and lack of penicillin components.  2. Clarithromycin-based Triple Therapy (if resistance to clarithromycin is not a concern):  - Clarithromycin: 500 mg twice daily  - Metronidazole: 500 mg three times daily  - PPI: such as omeprazole, 20 mg twice daily  3. Levofloxacin-based Triple Therapy:  - Levofloxacin: 500 mg once daily  - Amoxicillin: 1 g twice daily (if there is a specific cephalosporin allergy and not a general beta-lactam allergy)  - PPI: such as omeprazole, 20 mg twice daily  Note: In cases where the patient cannot tolerate amoxicillin, levofloxacin can be used with metronidazole instead.  Important Considerations  - Antibiotic Resistance: Local antibiotic resistance patterns should be considered when selecting a regimen. Clarithromycin resistance, in particular, is increasing and may impact the success of therapy.  - Duration of Therapy: Treatment is typically administered for 10-14 days, depending on the regimen and clinical guidelines.  - Follow-up Testing: Post-treatment testing (usually 4 weeks after completing therapy) is essential to confirm eradication of the infection.  Alternative Options  If standard therapies are contraindicated or if initial treatment fails, consider consulting a gastroenterologist for alternative therapies such as:  - Rifabutin-based triple therapy  - Dual therapy with high-dose PPI and amoxicillin (if penicillin allergy is not severe)  Adjunctive Therapy  - Probiotics: Some studies suggest that adding probiotics may help reduce side effects and improve eradication rates.  Managing Side Effects  - Metronidazole: May cause gastrointestinal upset and metallic taste.  - Tetracycline: Should be taken on an empty stomach and can cause photosensitivity.  Monitoring and Support  - Regular follow-up is essential to monitor for adverse effects and ensure adherence to the medication regimen.  - Educating patients on the importance of completing the full course of therapy is crucial for successful eradication and reducing resistance.  Always tailor the treatment to the individual patient's needs, considering their allergy profile and potential drug interactions. |
